# Managing Teachers' Job Attitudes: The Potential Benefits of Being a Happy and Emotional Intelligent Teacher

**DOI:** 10.3389/fpsyg.2021.661151

**Published:** 2021-05-04

**Authors:** María Angeles Peláez-Fernández, Sergio Mérida-López, Nicolás Sánchez-Álvarez, Natalio Extremera

**Affiliations:** ^1^Department of Social Psychology, Social Work, Social Anthropology and East Asian Studies, University of Málaga, Málaga, Spain; ^2^Department of Basic Psychology, University of Málaga, Málaga, Spain

**Keywords:** subjective happiness, emotional intelligence, job satisfaction, turnover intention, school teacher

## Abstract

According to the broaden-and-build theory of positive emotions, the frequency of positive emotions is associated with the development of positive attitudes, cognitions, and behaviors in organizational contexts. However, positive and negative attitudes at work might also be influenced by different personal and job resources. While emotional intelligence has been significantly associated with positive job attitudes and personal well-being, no studies have yet examined the joint role of teacher happiness and emotional intelligence in key teacher job attitudes. The present study assesses whether emotional intelligence interacts with levels of teacher happiness to jointly explain important teacher job attitudes (i.e., job satisfaction and turnover intention). A total sample of 685 teaching professionals (431 female) filled out a battery of scales including subjective happiness, emotional intelligence, job satisfaction, and turnover intention. Our results revealed that subjective happiness was significantly associated with both higher job satisfaction and lower turnover intention. Likewise, emotional intelligence was positively related to happiness and job satisfaction, and negatively related to turnover intention. Finally, interaction analysis showed the main effects of happiness and emotional intelligence in explaining teacher job attitudes. Beyond the main effects, the interaction effects of happiness and emotional intelligence were significant in predicting all teachers' job attitude indicators, even controlling for the effects of their sociodemographic variables. This work expands our knowledge about the role of teachers' positive emotions in the development of positive work attitudes, and also supports the inclusion of emotional skills in future teacher preparation programs as resources to facilitate work-related well-being.

## Introduction

Teaching is one of the most emotionally demanding jobs available (Travers, 2017; Iriarte Redín and Erro-Garcés, 2020). Teachers are required to strive to meet rules concerning which emotions to display in each of the diverse social interaction scenarios that arise in their workplace, many of which are characterized by highly emotionally demanding tasks. These emotional challenges often cause them distress, frustration, emotional exhaustion, and turnover intention (Travers, 2017; Granziera et al., 2021).

In the teaching context, the promotion of emotional competences and well-being would be of relevance to improve their coping skills and job satisfaction, as well as the quality of learning processes and the emotional development of the students (Jennings and Greenberg, 2009; Iriarte Redín and Erro-Garcés, 2020). There is evidence showing that high positive affectivity and subjective well-being buffers the impact of strain, stress, and boredom at work, leading to positive work outcomes (Benevene et al., 2019; De Stasio et al., 2019). Furthermore, several meta-analyses and systematic reviews show that positive affects predict an increase in job satisfaction (Boehm and Lyubomirsky, 2008), job performance (Judge et al., 2001), and work-related behaviors (Vacharkulksemsuk and Fredrickson, 2013). Thus, positive affectivity and subjective happiness have been underscored as key predictors of desirable work-related outcomes (Vacharkulksemsuk and Fredrickson, 2013).

Fredrickson's broaden-and-build theory of positive emotions (Vacharkulksemsuk and Fredrickson, 2013) proposes that frequent positive emotions prompted by subjective happiness at work influences teachers' work outcomes. For example, experiencing positive affects at work facilitates social and attitudinal abilities, allowing workers to enhance their personal resources, including sensitivity, positive attitudes, cognitions, and behaviors about their workplace (Vacharkulksemsuk and Fredrickson, 2013). Experiencing positive emotions may prompt teachers to build positive emotional connections with students, parents, and/or teaching staff members, leading to positive thinking and problem solving that allow the teachers to effectively deal with some of the most typical conflicts in the classroom (Isen, 2009). Thus, positive affect strengthens teachers' emotional enthusiasm and organizational well-being through increased and improved social interaction and positive teacher self-efficacy beliefs (Benevene et al., 2019; De Stasio et al., 2019).

Positive attitudes at work might operate with different personal resources and individual dispositions to facilitate work-related functioning (Vacharkulksemsuk and Fredrickson, 2013). According to the moderator model of EI (Côté, 2014), it is expected that the effects of personal dispositions (such as happiness) on organizational outcomes (such as teachers' turnover intention and job satisfaction) may vary according to levels of EI. In other words, it is expected that teachers with high subjective well-being and a greater ability to manage potential incidents and stressful situations related to the emotional scope of their teaching work would have more positive work attitudes (i.e., higher job satisfaction and lower turnover intention) compared with those with low subjective well-being and low emotional competences.

In this line, a recent study has found that subjective happiness together with compassion were significant predictors of increased teacher work engagement (De Stasio et al., 2019). One of those potential resources might be emotional intelligence (EI). From an ability perspective, EI is composed of different emotional skills such as the ability to perceive and express, use, understand, and regulate one's own emotions and those of others (Mayer et al., 2016). EI is typically linked to personal well-being and positive organizational outcomes including higher job satisfaction and lower turnover intention (Côté, 2014; Miao et al., 2017). In addition, there is theoretical and empirical support showing that EI moderates the relationship between contextual and dispositional factors, and work criteria (Côté, 2014). For instance, according to the meta-analysis conducted by Joseph and Newman (2010), the association between EI and job performance was stronger in emotionally demanding jobs.

Although there is empirical evidence that both positive effects and EI predict more positive organizational outcomes and greater personal well-being, no previous studies have examined the joint contribution of teachers' happiness and EI in affecting key teacher job attitudes. Understanding how both of these resources act in combination would help shed light on the mechanisms underlying the improvement of work variables and employees' well-being in a way that would contribute to enhancing both organizational variables and workers' quality of life. Thus, the purpose of the present study is to assess whether teacher happiness interacts jointly with EI to explain relevant teacher job attitudes. Following the broaden-and-build theory of positive emotions (Vacharkulksemsuk and Fredrickson, 2013) and the moderator model of EI (Côté, 2014), we state the following research hypothesis:

Hypothesis 1: *EI would moderate the relationship between happiness and job satisfaction (H1a), so that individuals with higher scores of happiness and higher levels of EI would report higher levels of job satisfaction*. *Moreover, EI would moderate the relationship between happiness and turnover intention (H1b), so that individuals with higher scores of happiness and higher levels of EI would report lower levels of turnover intention*.

## Materials and Methods

### Participants and Procedure

The study sample was comprised of 685 teaching professionals (62.9% female) working in childhood (15.2%), primary (36.1%), and secondary (48.8%) education in centers located in Southern Spain. The mean age was 44 years, with average teaching experience spanning 17 years. Most teachers (71.5%) had an indefinite contract at state-run institutions.

In line with previous studies, a student-recruited sampling method was used with the assistance of university students (e.g., Mérida-López et al., 2020). Teachers were informed that their participation was confidential and voluntary, and participants provided consent. Paper-and-pencil questionnaires were administered to the potential participants at school centers. The procedure was approved by the ethics committee of the University of Málaga (66-2018-H).

Regarding the instruments, well-validated measures were used. Happiness was measured with the Subjective Happiness Scale (SHS; Lyubomirsky and Lepper, 1999); this instrument is comprised of four items with a seven-point Likert-type scale. In this study, Cronbach's alpha was 0.78, which accords with results from the adaptation of the Spanish version of the scale (Extremera and Fernández-Berrocal, 2014). Overall EI was assessed with Wong and Law's Emotional Intelligence Test (WLEIS; Wong and Law, 2002). In line with prior research including the Spanish version of this instrument, Cronbach's alpha was 0.90 (Extremera et al., 2019). Job satisfaction was measured with an overall job satisfaction scale comprising five items with a seven-point Likert-type scale (Judge et al., 1998). In line with the values regarding the Spanish version, Cronbach's alpha was 0.77 in this study (Extremera et al., 2018). Finally, turnover intention was assessed with the Occupational Withdrawal Intentions Scale (Hackett et al., 2001) comprising three items; Cronbach's alpha was 0.94 in line with previous studies with Spanish teacher samples (Mérida-López et al., 2020).

### Analytical Strategy

First, Pearson correlations were used to test the associations among the main variables. Second, structural model was tested by structural equation modeling (SEM) to examine the main study hypotheses (H1a and H1b). The model was conceptualized by happiness, EI, and interaction product over job satisfaction or turnover intention. To reduce multicolinearity among interacting terms, we applied residual-centering procedure (Lance, 1988). Latent variables were defined by scale items following the full disaggregation approach (Bagozzi and Heatherton, 1994). A maximum likelihood approach was used, and to examine model fit indicators such as the Chi-square(χ^2^)/df, comparative fit index (CFI), Tucker and Lewis index (TLI), and the root mean square error of approximation (RMSEA) were considered (Schermelleh-Engel et al., 2003). Gender, age, teaching level, and teaching experience were entered as covariates so that any potential confounding effects on the dependent variables (i.e., job satisfaction and turnover intention) were controlled.

## Results

Table 1 illustrates the descriptive statistics, reliability coefficients, and correlations among the study variables. All the associations were significant and followed the expected direction. In sum, happiness was positively associated with EI and job satisfaction, and negatively related with turnover intention. On the other hand, EI was positively associated with job satisfaction and negatively linked with turnover intention. Finally, job satisfaction was negatively related with turnover intention.

**Table 1:** Descriptive statistics and bivariate correlations.

	**M (*SD*)**	**Alpha**	**1**	**2**	**3**	**4**
1. Happiness	5.38 (0.92)	0.78				
2. Emotional intelligence	5.53 (0.69)	0.90	0.52^**^			
3. Job satisfaction	5.62 (0.94)	0.77	0.44^**^	0.44^**^		
4. Turnover intention	1.79 (1.65)	0.94	−0.20^**^	−0.19^**^	−0.41^**^	

** *p < 0.01*.

**Figure 1:**
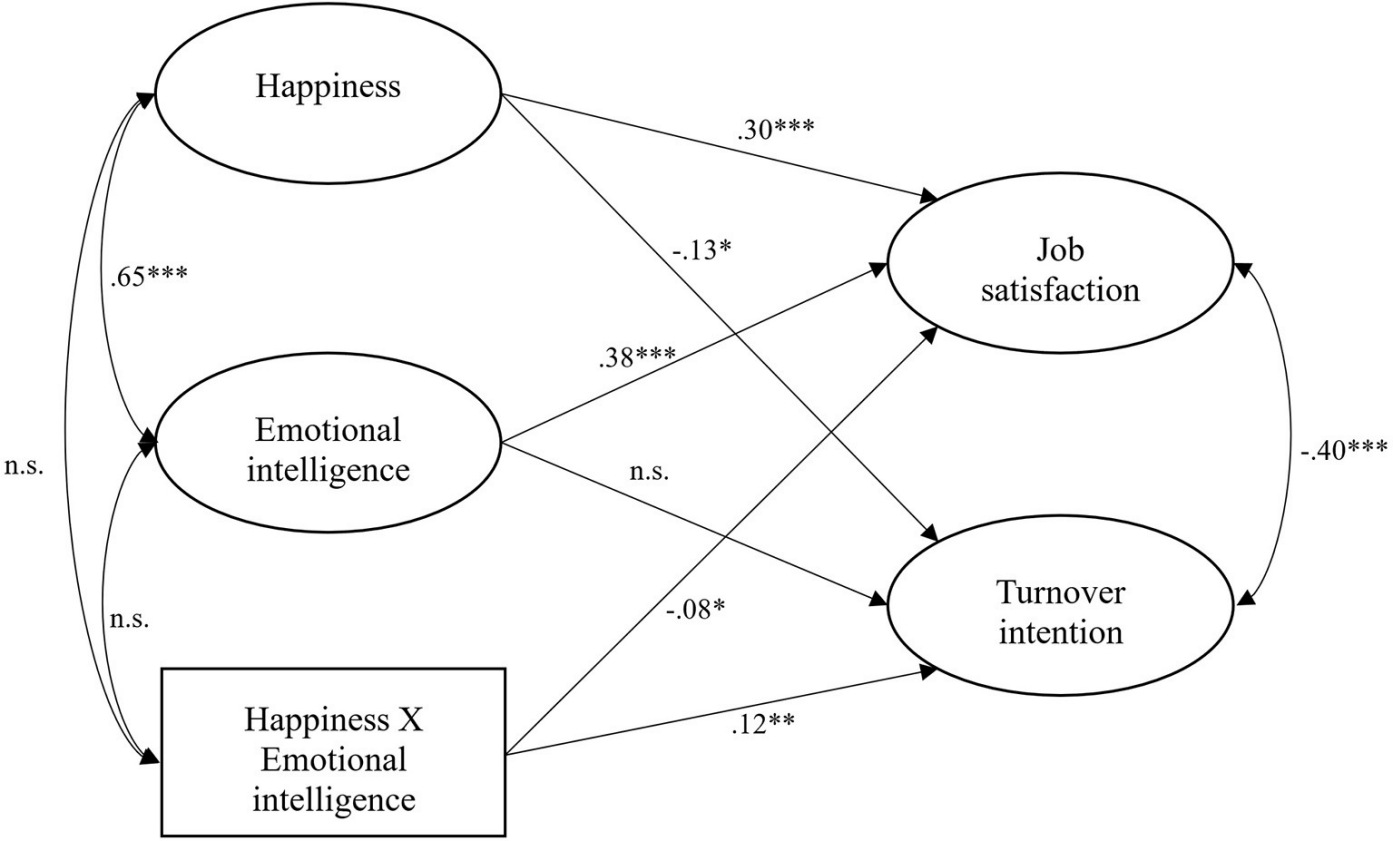
Standardized coefficient estimates of hypothesized SEM. Covariates (i.e., sex, gender, educational level, and teaching experience) and items included in the latent variables have been omitted in the figure representation; n.s., non-significant. *p < 0.05, **p < 0.01, ***p < 0.001.

SEM analysis using AMOS 20.0 was used to examine interaction effects model. Moderation model showed an excellent fit (χ^2^/df = 2.26, CFI = 0.95, TLI = 0.95, and RMSEA = 0.04). Figure 1 illustrates the main results. In sum, results indicated that the interaction between happiness and EI showed a significant effect on job satisfaction (b = −0.08, *p* = 0.028) and on turnover intention (b = 0.12, *p* = 0.002). In addition, happiness was associated with job satisfaction (b = 0.30, *p* < 0.001) and turnover intention (b = −0.13, *p* = 0.037), while the association of EI with job satisfaction (b = 0.38, *p* < 0.001) was significant, but association with turnover intention was not statistically significant. The percentages of variance explained were 41% for job satisfaction and 7% for turnover intention.

To illustrate the happiness × EI interactions for job satisfaction and turnover intention, two-way interactions with SEM results were computed (Preacher et al., 2006).

**Figure 2:**
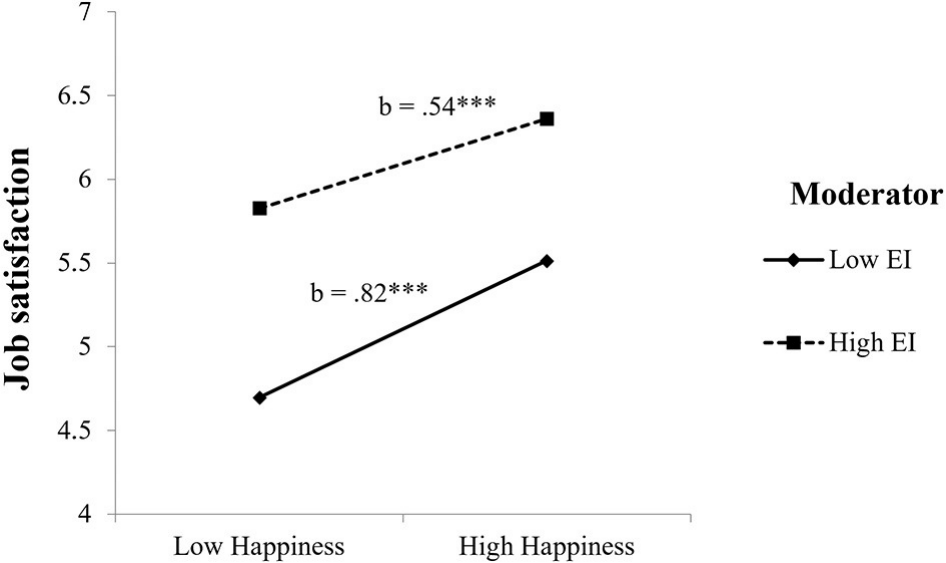
Interaction between EI and happiness on job satisfaction. ***p < 0.001.

As shown in Figure 2, the highest mean scores in job satisfaction were found among teachers reporting high (vs. low) happiness and high (vs. low) EI.

**Figure 3:**
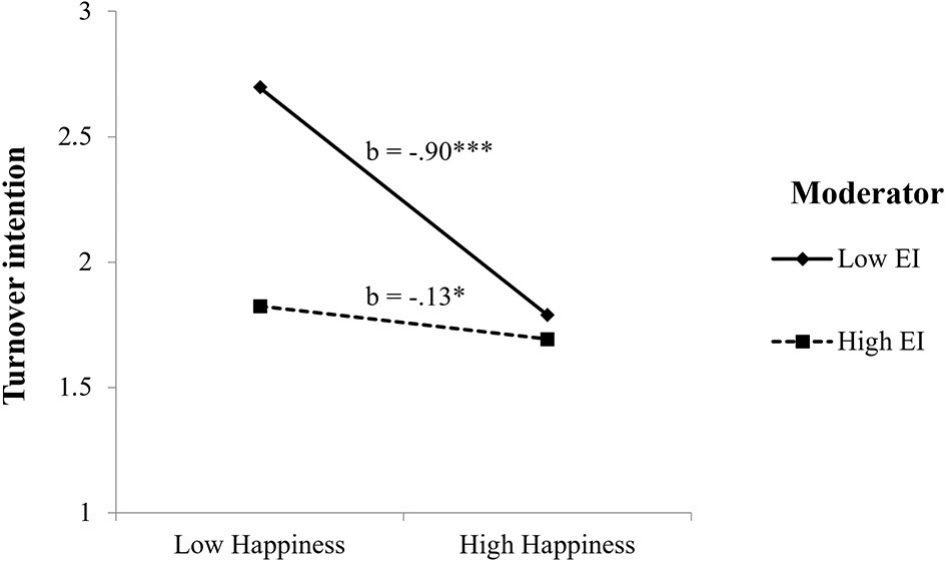
Interaction between EI and happiness on turnover intention. ***p < 0.001, *p < 0.05.

With regard to turnover intention, as Figure 3 shows, the results showed that the highest levels of turnover intention were found among those teachers with low (vs. high) happiness and low (vs. high) scores in EI.

## Discussion

The present study examined the link between teachers' subjective happiness and positive (i.e., job satisfaction) and negative (i.e., turnover intention) job attitudes, as well as the interactive role of EI in this association in a sample of Spanish teaching professionals. As expected, our results showed that both subjective happiness and EI were significantly associated with higher job satisfaction, as well as with lower turnover intention among teachers (Miao et al., 2017; De Stasio et al., 2019). Additionally, moderation analyses showed the main effects of happiness and EI in explaining teachers' job attitudes; in particular, happiness and EI predicted both higher job satisfaction and lower turnover intention. These results are in line with previous studies supporting that subjective well-being and EI predict higher job satisfaction and lower turnover intention (Boehm and Lyubomirsky, 2008; Vacharkulksemsuk and Fredrickson, 2013; Miao et al., 2017). Furthermore, and beyond the main effects, the interaction effects between happiness and EI were significant in predicting teachers' job attitudes, even when controlling for the effects of the sociodemographic and classic dimensions.

Regarding H1a, the results confirmed that happiness and EI interacted with each other to predict job satisfaction, so that teachers scoring high both in happiness and in EI reported the highest scores in job satisfaction. These results are similar to those of previous studies supporting the role of EI as a moderator variable modulating the effects of dispositional factors on work criteria (Côté, 2014). The current findings also support previous research showing that EI might act as a personal resource contributing to more positive work attitudes beyond the desirable effects of an intelligent and adaptive use of positive emotions on attitudes toward one's work (Tugade and Fredrickson, 2002). In our sample, teachers who were high in EI and happiness benefited from both resources and scored higher in job satisfaction than their counterparts with lower scores in these factors.

Concerning H1b, while non-significant main effects of EI on turnover intention were found, our results showed significant interactive effects of happiness with EI in predicting turnover intention. This may be explained in terms of the potential indirect mechanisms in the EI–job attitudes relationship, as suggested by Miao et al. (2017). Nonetheless, results indicated that those teachers with high scores in happiness and EI scored lower in turnover intention than their counterparts with low EI. These results are in agreement with Côté's moderator model (2014) and accord with prior empirical evidence on the moderating role of EI in the job attitudes–turnout intention (Mérida-López et al., 2020). Among teachers reporting high EI, there was a non-significant relationship between happiness and turnover intention. This finding may be explained by scrutinizing previous evidence suggesting that low levels of well-being and emotional deficits may relate to turnover intention; on the contrary, teachers with high EI may not necessarily display lower turnover intention, as this may depend on other factors such as the availability and quality of social and organizational resources at work (Miao et al., 2017).

Overall, our findings suggest that teachers experiencing high levels of happiness as well as perceiving themselves as emotionally intelligent may feel more capable of overcoming future teacher-related challenges and demands and that this may result in more positive attitudes toward their jobs and wider careers. The results from this study may add to the incipient literature on subjective well-being and dispositional factors as contributors to work-related well-being, as they show that happiness and EI may constitute beneficial and complementary resources that can influence teachers' perceptions of their work (De Stasio et al., 2019). Thus, these preliminary findings may contribute to developing comprehensive models integrating the JD-R theory and the broaden-and-build theory of positive emotions to achieve a better understanding of teachers' well-being (Granziera et al., 2021).

Our findings have practical implications for the prevention of negative attitudes and development of positive ones among at-risk teachers. Future teacher recruitment and retention programs might examine levels of subjective happiness as a key factor for developing positive job attitudes, and also assess potential deficits in EI as a potential risk factor for maintenance of unfavorable teacher job attitudes. Similarly, health promotion programs for teachers should incorporate workshops regarding socioemotional competences focused on the promotion of well-being and of effective emotion regulation strategies, which would help them cope with teaching stressors; this would reduce the mental health risks associated with negative attitudes toward school and teaching, while improving the quality of teaching (Jennings and Greenberg, 2009). Our results suggest that EI training programs would complement positive psychology interventions at work, which may result in increased positive attitudes toward teaching. Current findings underline the value of developing future programs focusing on teachers' positive emotions, as well as on their emotional skills, in order to improve job attitudes (Tugade and Fredrickson, 2002). For instance, these interventions may focus on positive emotions at work, as well as on the development of emotional skills, thus, fostering teachers' communication skills, their understanding of emotional dynamics and their ability to anticipate the emotional reactions of others, and to manage emotions more effectively during tense encounters in the classroom or with parents (Vacharkulksemsuk and Fredrickson, 2013; Iriarte Redín and Erro-Garcés, 2020). Therefore, empirically based programs focused on the training of socioemotional competences (Vesely-Maillefer and Saklofske, 2018) and on teachers' well-being (Fernandes et al., 2019) are recommended as direct and systemic elements of teachers' professional development that enable them to feel better equipped to meet the challenges of their work.

This study presents several limitations. First, as it was based on a cross-sectional design, the interpretations of the associations are limited. Future studies should include longitudinal research designs to examine the causal directions of these relationships. Second, our sample only included primary and secondary education teachers. Further studies should explore these relationships, providing data on the potential differences across teaching levels (Iriarte Redín and Erro-Garcés, 2020). Likewise, future studies should examine integrative models, testing the interplay of subjective well-being indicators, such as happiness, and personal resources, such as EI, with relevant organizational-level predictors of job attitudes (Granziera et al., 2021). Third, all variables were assessed using self-report measures, which might lead to problems of common method variance and possible biases implicit in the use of self-report instruments. Future studies should employ performance measures of EI or interviews.

Despite the aforementioned limitations, the present study increases our knowledge of the specific contribution of EI and positive emotions in teachers to the enhancement of well-being and work-related criteria; it also suggests the joint incorporation of both emotional abilities and positive activities for optimal well-being in preparation programs for future teachers, as key resources to increase positive and reduce negative attitudes toward their workplace.

## Data Availability Statement

The datasets presented in this article are not readily available because the dataset has been generated regarding a funded Research Project by Junta de Andalucia/FEDER funds (UMA18-FEDERJA-147). Requests to access the datasets should be directed to NE, nextremera@uma.es.

## Ethics Statement

The studies involving human participants were reviewed and approved by The Research Ethics Committee of the University of Malaga (66-2018-H). The patients/participants provided their written informed consent to participate in this study.

## Author Contributions

MP-F, SM-L, and NE created and organized the study and collected the data. SM-L, NS-A, and NE analyzed the data. MP-F and SM-L wrote the first draft. NE critically reviewed the manuscript and provided constructive comments. All authors contributed to the article and approved the submitted version.

## Conflict of Interest

The authors declare that the research was conducted in the absence of any commercial or financial relationships that could be construed as a potential conflict of interest.
